# Timely Advice

**Published:** 1900-07

**Authors:** 


### Abstracts and Selections.

### Timely Advice.

The following printed circular has been distributed to every family in San Antonio:

HEALTH DEPARTMENT,
SAN ANTONIO, TEXAS, May, 1900.

TO THE HEAD OF THE HOUSE:—The number of deaths amongst infants and children always increase during the summer months; it is the desire of the city health authorities to prevent this if possible, therefore your attention is asked to the following: Acute bowel diseases are the principal causes of deaths of infants and children under five years of age during hot weather.

These diseases are caused by

First—The weakening effects of the heat.

Second—Improper feeding and infected food.

Third—By unhealthy surroundings.

The food of infants under two years of age is chiefly milk. Milk after it is taken from the cow is soon infected or full of germ life. It is usually several hours old when delivered by milkmen. Milk pails or cans, though they may have been scalded or steamed, become quickly contaminated by exposure to the dust or dirt of cow pens.

The so-called "cholera infantum," inflammation of the stomach or bowels, or summer complaints of infants is due to infected milk, or improper food; in older children to green or decomposed fruits, vegetables, or melons.

Milk fed to infants should be scalded or boiled for one minute and then put in clean vessels, with closely fitting covers and placed on ice, in cold water, or buried in wet sand.

Boiling destroys germ life existing in milk, cold retards but does not, unless excessive, prevent milk from being contaminated by exposure to the air, hence the reason for keeping it in tightly covered vessels and cool after it is scalded or boiled.

The milk vessels, nursing botles, cups, nipples, spoons, etc., should be scalded or washed in soap and water each time before using, and milk should never be kept long or allowed to remain in nursing bottles or cups; it is just as easy to wash them immediately after using as it is to allow them to become foul, which they do very quickly, and then endanger the lives of infants. They are easier to clean and much safer if attended to at once. A little care on these lines is sure to save the lives of many infants.

No slops, filth, garbage, bath or dirty water should be allowed near or around your house; they are dangerous to health at all times and especially to infants and children during hot weather.

You may not have children but you can possibly aid in preventing deaths by helping to make known these recommendations.

F. PASCHAL,
Health Officer.

